# Trend of Bacterial Uropathogens and Their Susceptibility Pattern: Study of Single Academic High-Volume Center in Italy (2015–2019)

**DOI:** 10.1155/2021/5541706

**Published:** 2021-04-21

**Authors:** Enrica Serretiello, Veronica Folliero, Biagio Santella, Giuseppina Giordano, E. Santoro, Francesco De Caro, Pasquale Pagliano, Matteo Ferro, Silvana M. Aliberti, Mario Capunzo, Massimiliano Galdiero, Gianluigi Franci, Giovanni Boccia

**Affiliations:** ^1^Section of Microbiology and Virology, University Hospital “Luigi Vanvitelli”, Naples 80138, Italy; ^2^Department of Experimental Medicine, University of Campania “Luigi Vanvitelli”, Naples 80138, Italy; ^3^Department of Sanitary Hygiene and Evaluative Medicine U.O.C. Clinical and Microbiological Pathology, S. Giovanni di Dio and Ruggi D'Aragona University Hospital, “Scuola Medica Salernitana”, Largo Città di Ippocrate, Salerno 84131, Italy; ^4^Department of Medicine, Surgery and Dentistry “Scuola Medica Salernitana”, University of Salerno, Baronissi 84081, SA, Italy; ^5^Division of Urology, European Institute of Oncology, IRCCS, Milan, Italy

## Abstract

Urinary tract infections (UTIs) are a very widespread infection that can occur in disparate age range, in both sexes and in pregnancy/menopause state. Treatment of UTIs is difficult due to the emergence of antibiotic-resistant bacterial strains. The present study shows five years of data collected on patients admitted at the University Hospital “San Giovann di Dio e Ruggi d'Aragona” in Salerno, Italy. The investigation exhibits the incidence of the infection, of the gender, and of the age group affected, identifying the most representative bacteria involved, drawing their profile of antimicrobial resistance. Bacterial identification and antibiotic susceptibility testing were performed using the VITEK 2 system. Among the 46382 studied patients, 9896 (21.34%) and 36486 (78.66%) were positive and negative for microorganism growth, respectively. Of 9896 positive patients, 6158 (62.23%) females and 3738 (37.77%) males were identified. The highest incidence of positive subjects (56.66%) was recorded in the elderly (>61 years). 8431 (85.20%) uropathogens were Gram-negative, 1367 (13.81%) were Gram-positive, and 98 (0.99%) were *Candida* species (*Candida* spp.). *Escherichia coli (E. coli)* and *Enterococcus faecalis (E. faecalis)* were the most representative Gram-negative and Gram-positive strains, respectively. The Gram-negative bacteria most representative were highly resistant to ampicillin, whereas among the Gram-positive bacteria, *E. faecalis* was highly resistant to gentamicin and streptomycin high level synergy, and *Enterococcus faecium* (*E. faecium*) to ampicillin, ampicillin/sulbactam, and imipenem. This retrospective work investigates the local epidemiological trend in our university hospital in order to induce an increasingly targeted empirical therapeutic approach for the treatment of UTIs.

## 1. Introduction

Urinary tract infections (UTIs) represent widespread human microbial disorders involving any part of the urinary tract, such as the kidneys, bladder, urethra, and prostate [1]. UTIs are spread all over the world with a broad direct and indirect socioeconomic impact in the global population. Furthermore, these infections are associated with an important burden of morbidity and mortality and are second only to respiratory tract infections [2]. UTIs are estimated to affect approximately 150 million people each year in the world [3]. These infections cause more than 7 million medical visits and about 100,000 hospitalizations annually. Moreover, UTIs cost the world health system $ 6 billion a year [4]. According to the Infection Section of the European Association of Urology, UTIs are classified into complicated and uncomplicated infections. The first one is a condition that increases the risk to contract the infection, due to anatomical and functional abnormalities of the urinary tract, catheterization, or presence of an uphold disease [5]. The uncomplicated UTIs occur in healthy individuals without alterations of the urinary tract [6]. Based on the setting where the infection is contracted, UTIs are clustered in community or hospital-acquired infections. The community-acquired urinary tract infections are revealed in a community or within the first 48 hours of hospitalization [7]. The second one occurs 48 hours after hospitalization or 3 days after discharge [8]. UTI prevalence varies with age, gender, time of catheterization, hospital admission, and extensive antibiotic therapy [9]. Bacteria represent the leading cause of UTIs [3]. Gram-negative bacteria lead the 90% of UTIs, while only 10% of these infections are caused by Gram-positive bacteria [10, 11]. Prior studies identified *Escherichia coli (E. coli)* as the most frequent causative agent of UTIs, responsible for 65–90% of cases [12, 13]. However, the spectrum of uropathogens changes based on geographic region and clinic setting [9]. Diagnosis of UTIs consists of evaluating the patient's clinical signs and laboratory examination of urine [14]. The clinical signs depend on (i) urinary tract district; (ii) the uropathogens; (ii) immune state of the patient; (iii) age; and (iv) degree of the infection [15]. The most frequently encountered symptoms include (i) irregular and painful urination; (ii) back and abdominal pain; (iii) dysuria; and (iv) pyuria [16]. In some cases, positive urine culture can be associated with no symptoms [17]. Bacterial identification and antibiograms represent routine laboratory approaches [18]. The treatment of UTI occurs through the use of broad-spectrum antibiotics [19]. Inappropriate use of these antibiotics has inevitably led to a massive increase in antimicrobial resistance (AMR). Due to the emergence of AMR, UTI patients may develop negative outcomes after antibiotic treatment and encounter serious clinical complications [20, 21]. Multidrug-resistant bacteria and high prevalence of UTIs underline the need to better understand the causative agents of UTIs and their antibiotic resistance profiles. The purpose of the present study was to evaluate the bacterial pathogens, involved in UTIs, and their antimicrobial susceptibility pattern in patients admitted to the San Giovanni di Dio e Ruggi d'Aragona Hospital. This is crucial to set new guidelines for the better choice of empiric antibiotic treatment in our hospital.

## 2. Materials and Methods

### 2.1. Sample Collection

In the last five years (between January 2015 and December 2019) a total of 46382 urinary samples were collected from patients admitted to the University Hospital “San Giovanni di Dio e Ruggi d'Aragona.” In particular, midstream specimens of urine (MSU) were consigned to microbiological laboratory and processed as described below.

### 2.2. Inclusion and Exclusion Criteria

The samples tested met the following inclusion criteria: (i) patients aged 0 to 99; (ii) clinical evidence of one or more symptoms of UTI, such as dysuria, frequency, hesitation, urgency, and pain, was recorded in patients included in the analysis; and (iii) bacterial count value must be greater than equal to of 10^5^ CFU/mL in the urine at mid flow to be deemed culture positive. Exclusion criteria were as follows: (i) patients with urinary catheter were excluded by the analysis; and (ii) bacterial count less than 10^4^ CFU/mL was evaluated culture negative in medium flow urine.

### 2.3. Bacterial Culture

The samples were sown using the automated plating system ALFRED60 (Alifax) in accordance with the manufacturer's recommendations on CHROMID® CPS® Elite plate (Biomerix Corporation, France) and incubated for 24 hours at 37°C. If the growth of two or more bacterial species was observed, the samples were regarded as contaminated (exclusion criteria). Urinary cultures were negative if the number of colony forming units per mL (CFU/mL) was less than 10^3^ (exclusion criteria). Bacteriuria was defined by the number greater than 10^5^ CFU/mL and by a monomorphic growth (inclusion criteria). In this instance, bacterial identification and antimicrobial sensitivity test were executed.

### 2.4. Bacterial Identification and Antibiotic Susceptibility Test

After each plate examination, bacterial identification and antimicrobial susceptibility test were performed via technology Vitek 2 (BioMe'rieux, France), following the manufacturer's recommendations. The results of antimicrobial susceptibility were interpreted as “susceptible,” “resistant,” or “intermediate” according to EUCAST guidelines and obtained after 16 h of incubation. In particular, the antimicrobial molecules examined in this study were amikacin, ampicillin, ampicillin/sulbactam, amoxicillin/clavulanic acid, cefepime, cefotaxime, ceftazidime, ciprofloxacin, ertapenem, fosfomycin c/G6P, gentamicin and gentamicin high level synergy, imipenem, levofloxacin, linezolid, meropenem nitrofurantoin, norfloxacin, piperacillin/tazobactam, streptomycin high level synergy, tigecycline, teicoplanin, trimethoprim/sulfamethoxazole, and vancomycin, in accordance with the typology of bacteria analysed. Antibiotics were selected in agreement with EUCAST guidelines.

### 2.5. Data Analysis

Data analysis was performed using IBM Statistical Package for Social Sciences Version 22.00 (IBM SPSS Inc., USA). The significance of the antibiotics resistance trend during the five years was analysed using the chi-framework (http://www.spss.com). *p* values < 0.05 were considered statistically significant.

### 2.6. Ethical Consideration Statement

Ethics approval from the Human Research Ethics Committee was not required for this study. Our study exploited laboratory management data, collected from databases. This represents a retrospective study and is not directly associated with patients.

### 2.7. Limitations

Although our exposed data are very precious in a context where information on antimicrobial resistance of UTIs strains is limited, some limitations should be considered. First, our study was limited to a single clinical service. Prospective studies involving multiple hospitals are needed to establish criteria for treatment options to be extended beyond our hospitals. Second, although basic patients' demographics and clinical signs are commonly available, other important information such as clinical details, hospitalization period, treatment received, and clinical outcomes is often unavailable.

## 3. Results

### 3.1. Incidence and Gender/Age Group Distribution of UTIs in Urinary Specimens

A total of 46382 urine samples were examined. Patients between the ages of 1 and 99 and of both genders were included in the study. UTI diagnosis was based on the patient's clinical symptomatology, as well as on the presence of leukocytes and bacteria in the urine samples investigated. Of 46382 samples, 9896 (21.34%) were positive for growth of pathogenic strains, while 36486 (78.66%) were negative; the incidence of samples positive and/or negative for every year and in the total five years of analysis is reported (Table 1).

Among the total positive pathogenic isolates, the Gram-positives were 1367 (13.81%), while the Gram-negatives were the most representative with 8431 (85.20%) isolated strains, and 98 (0.99%) were *Candida* species. Data were analysed in order to investigate the incidence of UTI for gender (Table 2). Female gender was more exposed to the infection with 6158 (62.23%) positive cultures, while 3738 (37,77%) were the male positive cultures (Table 2).

Analysis of age infection distribution showed that the major incidence was recorded in the elderly (>61 years) (56.6%), followed by late adulthood (46–60 years) (18.25%), young adults (19–45 years) (15.79%), infants (<1 year) (7.04%), early childhood (2–5 years) (0.91%), late childhood (6–12 years) (0.75%), and adolescents (13–18 years) (0.60%) (Table 3). The female to male ratio was higher in the age group 19–45 years (F/M = 3), while it was lower in the age group <1 year (F/M = 0.73).

**Figure 1:**
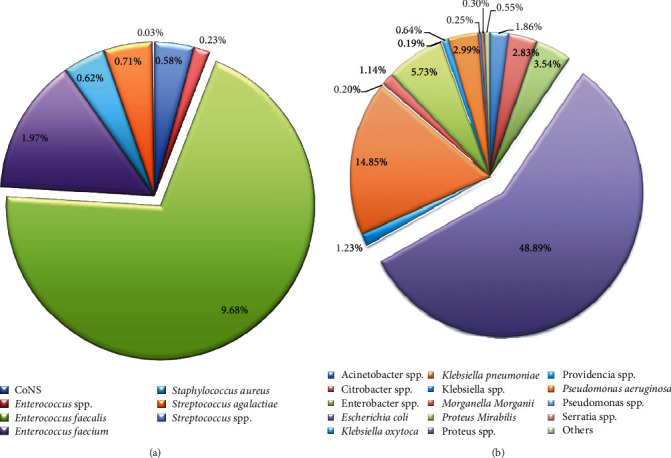
Prevalence of Gram-positive (a) and Gram-negative (b) on the total microorganisms isolated from urine culture samples. “Others” represent genera or species less representative, thus they were merged.

The positive patients were admitted to the department of High-Risk Pregnancy and Prenatal Diagnosis, Urology, Paediatrics, Neonatology, Nephrology, Infectious Disease, Kidney Transplant Center, Obstetrician, Gynaecology, General Medicine Women, Neurology, Gastroenterology, Emergency Medicine, and others. Bacterial species were isolated and identified by 9896 positive cultures. Analysing the Gram-positive bacteria incidence (13.81%), our data reported that *Enterococcus faecalis (E. faecalis)* was the most representative isolated bacterium (9.68%). Lower percentages of incidence resulted for *Enterococcus faecium (E. faecium)* (1.97%), *Streptococcus agalactiae* (*S. agalactiae)* (0.71%), *Staphylococcus aureus (S. aureus)* (0,62%), *Staphylococcus* coagulase negative (CoNS) (0,58%), *Enterococcus* spp. (0,23%), and *Streptococcus* spp. (0.03%) (Figure 1(a)). The highest percentage of isolated bacteria belonged to Gram-negative bacteria (85.20). In particular, *E. coli* resulted to be the most frequently isolated bacterium (48.89%), followed by *Klebsiella pneumoniae* (*K. pneumoniae)* (14.85%), *Proteus mirabilis* (*P. mirabilis)* (5.73), *Enterobacter* species (*Enterobacter* spp.) (3.54%), *Pseudomonas aeruginosa* (*P. aeruginosa)* (2.99%), Citrobacter spp. (2.83%), *Acinetobacter* spp. (1.86%), *Klebsiella oxytoca (K. oxytoca)* (1.23%), *Morganella morganii (M. morganii*) (1.14%), and *Providencia* spp. (0.64%). Very low % was observed for *Serratia* spp. (0.30%), *Pseudomonas* spp. (0.25%), *Klebsiella* spp. (0.20%), and *Proteus* spp. (0.19%), and we called all other species that we identify in very low abundance “others” (*Achromobacter* spp., *Aeromonas* spp., *Burkholderia gladioli*, *Cedecea* spp., *Edwardsiella tarda*, *Escherichia vulneris, Hafnia alvei, Kluyvera intermedia, Moellerella wisconsensis, Moraxella* spp., *Pasteurella pneumotropica*, *Raoultella* spp., *Salmonella* spp., *Shigella dysenteriae, Sphingomonas paucimobilis, Stenotrophomonas maltophilia,* and *Yersinia pseudotuberculosis*) with a % of 0.55 (Figure 1(b)).

**Figure 2:**
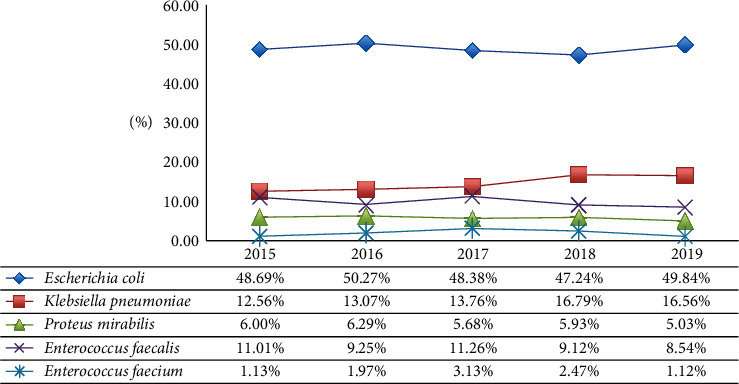
Trend incidence of the most representative Gram-positive and Gram-negative uropathogens isolated from urine samples during the analysed five years.

On the other hand, Figure 2 shows the incidence variation of the most representative Gram-positive and Gram-negative uropathogens found during the analysed five years. The prevalence of each UTIs strain remained approximately constant through time. *E coli* is the dominant effective agent in our hospital over the period studied. A significant trend in *E. coli* (*p* value = 0.039) and *P. mirabilis* (*p* value = 0.047) was detected.

### 3.2. Uropathogens' Antimicrobial Resistance Profile

**Figure 3:**
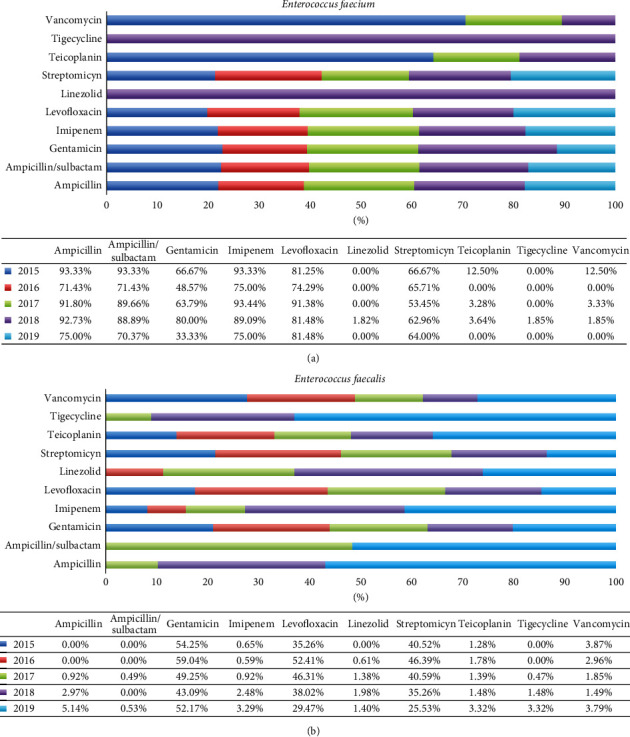
Antimicrobial susceptibility pattern of the most representative Gram-positive bacteria. The percentage of antibiotic resistance of (a) *Enterococcus faecium* and (b) *Enterococcus faecalis* is reported for each year investigated in the study. Gentamicin and streptomycin are both used as “high level synergy.”

**Figure 4:**
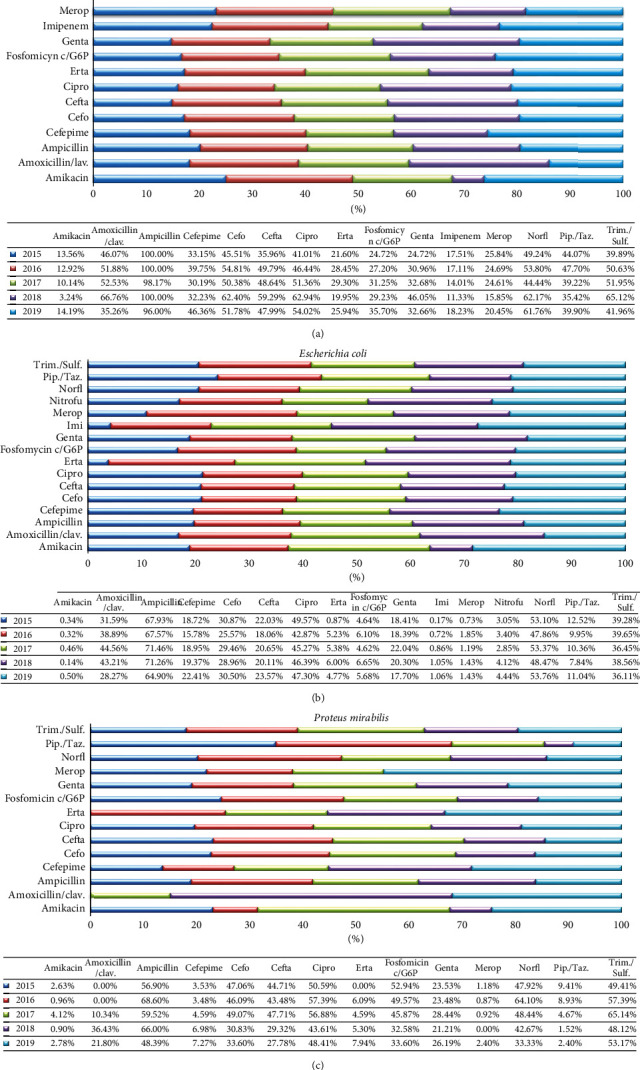
Antimicrobial susceptibility pattern of the most representative Gram-positive bacteria. The percentage of antibiotic resistance of (a) *Klebsiella pneumoniae*, (b) *Escherichia coli*, and (c) *Proteus mirabilis* is reported for each year investigated in the study. Abbreviations: clav. = clavulanic acid; Cefo = cefotaxime; Cefta = ceftazidime; Cipro = ciprofloxacin; Erta = ertapenem; Genta = gentamicin; Merop = meropenem; Nitrofu = nitrofurantoin; Norfl = norfloxacin; Pip./Taz. = piperacillin/tazobactam; Trim./Sulf. = trimetoprim/sulfamethoxazole.

In addition to the main bacteria representative identification among the total positive urine samples, the present study exhibits their relative antimicrobial resistance profile too. In particular, the most representative Gram-positive (*E. faecium and E. faecalis*) and Gram-negative (*K. pneumoniae, E. coli*, *and P. mirabilis*) antimicrobial resistance profile is reported in Figures 3 and 4 , respectively.

The data commented in the text below refer to the percentages of the total five years, indicated as 2015–2019 in the Supplementary Tables (S1–S5). Among the Gram-positive identified bacteria in urine samples, *E. faecium* turned out to be the most resistant strain against several antibiotics, such as ampicillin, ampicillin/sulbactam, imipenem, and levofloxacin (86.08%, 83.60%, 86.15%, and 83.16%, respectively). Very low % of resistance was found against linezolid (0.52%), tigecycline (0.55%), and vancomycin (2.58%). Total resistance to ampicillin was steadily high, ranging between 71.43% and 93.33% throughout the study period (*p* value = 0.03). Resistance to gentamicin decreased from 66.67% to 48.57% between 2015 and 2016, increased up to 80% in 2018, and finally decreased to 33.33% in 2019 (*p* value = 0.03). The levels of resistance to streptomycin were almost constant over the study period (64.83%), and a decrease of up to 53.45% in 2017 was observed (*p* value = 0.045) (Figure 3(a)).

*E. faecalis*, the first Gram-positive in order of abundance, seems to be very responsive to several antibiotics such as ampicillin/sulbactam, with a 0.22% of resistance, linezolid and tigecycline (1.16%), imipenem (1.68%), teicoplanin (1.88%), ampicillin (1.99%), and vancomycin (2.73%). *E. faecalis* resistance for the antibiotics gentamicin high level synergy, levofloxacin, and streptomycin high level synergy showed a middle value (51.10%, 40.24%, and 37.37%, respectively). An increase in ampicillin (*p* value = 0.03), tigecycline (*p* value = 0.02), and imipenem (*p* value = 0.04) resistance of up to 5.14%, 3.32%, and 3.29% was observed over the years, respectively. Resistance levels to ampicillin/sulbactam were constant over the years investigated (*p* value = 0.0005). Resistance to streptomycin showed a significant decrease from 40.52% in 2015 to 25.53% in 2019. Levofloxacin resistance level increased from 35.26% to 52.41% between 2015 and 2016 and decreased up to 29.47% in 2019 (*p* value = 0.003). Resistance to gentamicin showed an increase from 54.25% to 59.04% between 2015 and 2016, a decrease up to 43.09% in 2018, and an increase to 52.17% in 2019 (*p* value = 0.004) (Figure 3(b)).

With regard to Gram-negative, *K. pneumoniae* showed a profile of high resistance for several antibiotics. In particular, the highest percentage (98.99%) resulted for ampicillin, followed by a percentage of about 50% for amoxicillin/clavulanic acid, cefotaxime, ceftazidime, ciprofloxacin, norfloxacin, and trimethoprim/sulfamethoxazole (51.5%, 53.96%, 49.79%, 52.95%, 54.40%, and 50.83 %, respectively). Lower strength rates were found for use of cefepime, ertapenem, fosfomycin c/C6P, gentamicin, imipenem, meropenem, and piperacillin/tazobactam (36.74%, 30.47%, 34.82%, 15.39%, 21.39%, and 40.45%, respectively). The lowest percentage of resistance was recorded for amikacin, with a resistance of 10.35%. Resistance level to amikacin was almost constant over the time (12.7%), and a decrease of up to 3.24% in 2018 was observed (*p* value = 0.02). The resistance rates to ampicillin (98.83%) and imipenem (15.64%) were constant throughout the study period (*p* value = 0.02). Resistance to amoxicillin increased from 46.07% to 66.76% between 2015 and 2018 and decreased up to 35.26% in 2019 (*p* value = 0.01). An increase in ampicillin resistance from 33.15% to 39.75% between 2015 and 2016, a decrease up to 32.23% in 2018, and an increase up to 46.36% were observed (*p* value = 0.03). The resistance to piperacillin was constant over time, ranging between 35.92% and 47.7% (*p* value = 0.002) (Figure 4(a)). *E. coli* showed high resistance for ampicillin (69.00%). Furthermore, this strain showed a 21% higher resistance rate to norfloxacin, ciprofloxacin, trimethoprim/sulfamethoxazole, amoxicillin/clavulanic acid, cefotaxime, and ceftazidime. For cefepime and gentamicin, lower percentages than the above were found (19.28% and 19.33%). *E. coli* showed the lowest % of resistance for amikacin (0.36%), ertapenem (4.69%), fosfomycin c/C6P (5.62%), nitrofurantoin (3.66%), meropenem (1.36%), and piperacillin/tazobactam (10.22%). The rates of resistance to amikacin (*p* value = 0.03), norfloxacin (*p* value = 0.003), gentamicin (*p* value = 0.047), ciprofloxacin (*p* value = 0.03), ceftazidime (*p* value = 0.04), cefotaxime (*p* value = 0.04), cefepime (*p* value = 0.005), and ampicillin (*p* value = 0.0005) were constant over time (Figure 4(b)). *P. mirabilis*, the third strain for percentage of incidence, resulted very sensitive for meropenem, amikacin, piperacillin/tazobactam, ertapenem, and cefepime with a resistance of 1.06%, 2.22%, 4.99%, 5.14%, and 5.29% respectively. An intermediate value of resistance about 20% to 60% was observed for amoxicillin/clavulanic acid, ampicillin, cefotaxime, ceftazidime, ciprofloxacin, fosfomycin c/C6P, gentamicin, norfloxacin, and trimethoprim/sulfamethoxazole (18.94%, 62.12%, 40.46%, 37.68%, 51.06%, 41.87%, 24.51%, 50.73%, and 54.58%, respectively). A significant decline in fosfomycin resistance from 52.94% to 33.6% was detected over the study period (*p* value = 0.02). The levels of resistance to meropenem (*p* value = 0.04), ciprofloxacin (*p* value = 0.05), and cefepime (*p* value = 0.02) were stable over time. A significant reduction in resistance rates to cefotaxime from 47.06% to 33.6% (*p* value = 0.05) and ceftazidime from 44.7% to 27.8% (*p* value = 0.05) was detected. In contrast, a significant increase in amoxicillin resistance from 0% to 21.8% was observed (*p* value = 0.03). Ampicillin resistance ranged from 48.39% to 68.61% throughout the study period (*p* value = 0.05) (Figure 4(c)).

## 4. Discussion

The present paper shows data collected about patients of all ages and both sexes admitted at the University Hospital “San Giovanni di Dio e Ruggi d'Aragona” in Salerno, Italy, from January 2015 to December 2019. Analysis of our findings showed that 21.34% resulted positive for microorganism's growth. Opposite results were found in Bushenyi District in Uganda (32.2%) and at National Hospital Abuja in Nigeria (13.1%) [15, 22]. The largest number of positive patients belonged to the female gender (62.23%). Their high predisposition to contract infection is due to their genital anatomy [10]. As regards age groups, highest prevalence was found in the elderly group (56,6%), due to several factor, such as urinary tract abnormalities, disability, decreased immune response, and prostate disorders in men and hormonal changes in women [10, 23, 24]. Among detected UTI strains, 13.81% were Gram-positive, 85.20% were Gram-negative and 0.99% were *Candida* spp. *E. coli* (48.89%) was the most represented Gram-negative strain, followed by *K. pneumoniae* (14.85%) and *P. mirabilis* (5.73%). Among Gram-positive strains, *E. faecalis* was the most frequently isolated strain (9.68%), followed by *E. faecium* (1.97%) and *S. agalactiae* (0.71%). The prevalence of identified bacteria was comparable to other reports in different countries. In Colombia, *E. coli* was involved in 39.7% UTI cases, followed by *Enterococcus* spp. (11.5%) [2]. Moreover, in a Chinese study, *E. coli* and *E. faecalis* were the most isolated uropathogens, causing 66.01 and 5.91% of UTI infections [25]. Bacterial resistance profile showed that *E. faecium* was the most resistant Gram-positive strain. This uropathogen exhibited a high percentage of resistance for ampicillin, ampicillin/sulbactam, imipenem, levofloxacin, and streptomycin high level synergy. On the other hand, E. faecium showed a relevant sensitivity to linezolid, tigecycline, vancomycin, and teicoplanin. More sensitive than *E. faecium* was *E. faecalis*, which showed very low percentage of resistance to ampicillin, ampicillin/sulbactam, imipenem, linezolid, tigecycline, teicoplanin, and vancomycin. A prominent data was the significant reduction in streptomycin resistance levels from 40.52% to 25.53%. Similar data were obtained from a study conducted at University of Campania “Luigi Vanvitelli” in Italy [10]. Comparable results were observed from a study conducted in India by Bharti et al. They reported a 92% higher sensitivity rate to linezolid, vancomycin, and teicoplanin [26]. Concerning Gram-negative bacterial strains, *K. pneumonia* was identified as the most resistant uropathogen. This strain reported a 96% higher resistance rate to ampicillin. Its inappropriate use represents a plausible cause of the high ampicillin resistance rate over time. A percentage of resistance of less than 30% was found for amikacin, imipenem, ertapenem, and meropenem. Strains of *E. coli* also showed low resistance to carbapenems and amikacin. The prevalence of ESBL (extended spectrum beta lactamase) for *E. coli* and *K. pneumoniae* strains was monitored. During the studied period, a total of 3206 *E. coli* were detected, including 30.75% which showed the ESBL phenotype. Comparable data were obtained for *K. pneumoniae*, where 35.43% were ESBLs (data not shown). The 3rd most frequently isolated Gram-negative bacteria were *P. mirabilis*. This strain showed a high resistance versus ampicillin, ciprofloxacin, norfloxacin, and trimethoprim/sulfamethoxazole. The lowest resistance resulted for the antibiotics amikacin cefepime, ertapenem, meropenem, and piperacillin/tazobactam. An important reduction of fosfomycin resistance from 52.94% to 33.6% was found in study period. The most represented Gram-negative bacteria exhibited a high rate of resistance to ampicillin, according to the Campania region antibiotic-resistant report. Comparable results were reported in Ethiopia, where 78% of Gram-negative strains were resistant to ampicillin [27]. Lower ampicillin resistance rates were observed in India (*R* > 35%) [28]. Our data demonstrated that carbapenems could be used for the treatment of infections caused by Gram-negative strains. Studies conducted in our region reported that resistance to carbapenem drugs was the lowest compared to other antibiotics. Otherwise, linezolid and glycopeptides may be the appropriate treatments for Gram-positive strains, in accordance with our regional reports [29]. Aware of the problem of antimicrobial resistance in hospital settings, our study will influence the choice of empirical therapy for UTIs.

## 5. Conclusions

The antimicrobial susceptibility profiles of the strains most represented in the cases of UTIs have focused attention on the poor control and management of UTIs. We suggest that the choice of empirical antibiotic therapy should be based on knowledge of the localized epidemiological trend. This study reports information on the current situation in our university hospital, in order to establish guidelines for the correct use of antibiotics.

## Figures and Tables

**Table 1:** Number of urinary specimens analysed by the year.

	Years					
Samples	2015	2016	2017	2018	2019	Total
Positive (N.)	1417	1828	1918	2227	2506	9896
(%)	16.27	21.32	22.20	21.77	24.50	21.34
Negative (N.)	7293	6745	6722	8005	7721	36486
(%)	83.73	78.68	77.80	78.23	75.50	78.66
Total (N.)	8710	8573	8640	10232	10227	46382

**Table 2:** Percentage of Gram-positive and Gram-negative bacteria and *Candida* spp. isolated in urine specimens collected in the period 2015–2019.

Microorganisms isolated	*n*. (%)
Gram-positive bacteria	1367 (13.81)
Gram-negative bacteria	8431 (85.20)
*Candida* spp.	98 (0.99)
Gender	*n*. (%)
Female	6158 (62.23)
Male	3738 (37.7)

**Table 3:** Incidence of positivity specimens in the analysed age groups and among the genders.

Age group (years)	Gender *n*. (%)		
	Female	Male	Total
<1	294 (4.77)	403 (10.78)	697 (7.04)
2–5	53 (0.86)	37 (0.99)	90 (0.91)
6–12	48 (0.78)	26 (0.70)	74 (0.75)
13–18	36 (0.58)	23 (0.62)	59 (0.60)
19–45	1172 (19.03)	391 (10.46)	1563(15.79)
46–60	1047 (17.00)	759 (20.30)	1806(18.25)
>61	3508 (56.97)	2099(56.15)	5607(56.66)

## Data Availability

No data were used to support this study.

## Supplementary Materials

Supplementary Materials Supplementary Table 1: antimicrobial susceptibility pattern of *Enterococcus faecium*. Supplementary Table 2: antimicrobial susceptibility pattern of *Enterococcus faecalis*. Supplementary Table 3: antimicrobial susceptibility pattern of *Klebsiella pneumoniae*. Supplementary Table 4: antimicrobial susceptibility pattern of *Escherichia coli*. Supplementary Table 5: antimicrobial susceptibility pattern of *Proteus mirabilis*.
