# Supplementary material for: Trend of Bacterial Uropathogens and Their Susceptibility Pattern: Study of Single Academic High-Volume Center in Italy (2015–2019)
Source: Int J Microbiol. 2021 Apr 21;2021:5541706. doi: 10.1155/2021/5541706 (PMC8116166; doi:10.1155/2021/5541706)
Supplement: Supplementary Materials — Supplementary Table 1: antimicrobial susceptibility pattern of Enterococcus faecium. Supplementary Table 2: antimicrobial susceptibility pattern of Enterococcus faecalis. Supplementary Table 3: antimicrobial susceptibility pattern of Klebsiella pneumoniae. Supplementary Table 4: antimicrobial susceptibility pattern of Escherichia coli. Supplementary Table 5: antimicrobial susceptibility pattern of Proteus mirabilis. [file 5541706.f1.docx]

| ***Enterococcus faecium*** | **2015** | | | **2016** | | | **2017** | | | **2018** | | | **2019** | | | **2015-2019** | | |
| --- | --- | --- | --- | --- | --- | --- | --- | --- | --- | --- | --- | --- | --- | --- | --- | --- | --- | --- |
| **ANTIBIOTIC** | **Assays n.** | **I%** | **R%** | **Assays n.** | **I%** | **R%** | **Assays n.** | **I%** | **R%** | **Assays n.** | **I%** | **R%** | **Assays n.** | **I%** | **R%** | **Assays n.** | **I%** | **R%** |
| *Ampicillin* | 15 | 0,00 | 93,33 | 35 | 2,86 | 71,43 | 61 | 1,64 | 91,80 | 55 | 0,00 | 92,73 | 28 | 7,14 | 75,00 | 194 | 2,06 | 86,08 |
| *Ampicillin/sulbactam* | 15 | 0,00 | 93,33 | 35 | 0,00 | 71,43 | 58 | 3,45 | 89,66 | 54 | 0,00 | 88,89 | 27 | 3,70 | 70,37 | 189 | 1,59 | 83,60 |
| *Gentamicin* | 15 | 0,00 | 66,67 | 35 | 0,00 | 48,57 | 58 | 0,00 | 63,79 | 50 | 0,00 | 80,00 | 6 | 0,00 | 33,33 | 164 | 0,00 | 64,63 |
| *Imipenem* | 15 | 0,00 | 93,33 | 36 | 13,89 | 75,00 | 61 | 0,00 | 93,44 | 55 | 0,00 | 89,09 | 28 | 0,00 | 75,00 | 195 | 2,56 | 86,15 |
| *Levofloxacin* | 16 | 0,00 | 81,25 | 35 | 0,00 | 74,29 | 58 | 0,00 | 91,38 | 54 | 0,00 | 81,48 | 27 | 0,00 | 81,48 | 190 | 0,00 | 83,16 |
| *Linezolid ** | 14 | 0,00 | 0,00 | 36 | 0,00 | 0,00 | 60 | 0,00 | 0,00 | 55 | 0,00 | 1,82 | 28 | 0,00 | 0,00 | 193 | 0,00 | 0,52 |
| *Streptomicyn* | 15 | 0,00 | 66,67 | 35 | 0,00 | 65,71 | 58 | 0,00 | 53,45 | 54 | 0,00 | 62,96 | 25 | 0,00 | 64,00 | 187 | 0,00 | 60,96 |
| *Teicoplanin ** | 16 | 0,00 | 12,50 | 36 | 0,00 | 0,00 | 61 | 0,00 | 3,28 | 55 | 0,00 | 3,64 | 28 | 0,00 | 0,00 | 196 | 0,00 | 3,06 |
| *Tigecycline ** | 15 | 0,00 | 0,00 | 33 | 0,00 | 0,00 | 54 | 0,00 | 0,00 | 54 | 0,00 | 1,85 | 27 | 0,00 | 0,00 | 183 | 0,00 | 0,55 |
| *Vancomycin ** | 16 | 0,00 | 12,50 | 36 | 0,00 | 0,00 | 60 | 0,00 | 3,33 | 54 | 0,00 | 1,85 | 28 | 0,00 | 0,00 | 194 | 0,00 | 2,58 |

| ***Enterococcus faecalis*** | **2015** | | | **2016** | | | **2017** | | | **2018** | | | **2019** | | | **2015-2019** | |  |
| --- | --- | --- | --- | --- | --- | --- | --- | --- | --- | --- | --- | --- | --- | --- | --- | --- | --- | --- |
| **ANTIBIOTIC** | **Assays n.** | **I%** | **R%** | **Assays n.** | **I%** | **R%** | **Assays n.** | **I%** | **R%** | **Assays n.** | **I%** | **R%** | **Assays n.** | **I%** | **R%** | **Assays n.** | **I%** | **R%** |
| *Ampicillin ** | 153 | 0,65 | 0,00 | 169 | 0,59 | 0,00 | 217 | 0,46 | 0,92 | 202 | 0,00 | 2,97 | 214 | 0,00 | 5,14 | 955 | 0,31 | 1,99 |
| *Ampicillin/sulbactam ** | 153 | 0,00 | 0,00 | 166 | 0,00 | 0,00 | 203 | 0,00 | 0,49 | 191 | 0,52 | 0,00 | 190 | 0,00 | 0,53 | 903 | 0,11 | 0,22 |
| *Gentamicin* | 153 | 0,00 | 54,25 | 166 | 0,00 | 59,04 | 201 | 0,00 | 49,25 | 181 | 0,00 | 43,09 | 23 | 0,00 | 52,17 | 724 | 0,00 | 51,10 |
| *Imipenem ** | 153 | 0,00 | 0,65 | 169 | 0,00 | 0,59 | 217 | 0,46 | 0,92 | 202 | 0,00 | 2,48 | 213 | 0,00 | 3,29 | 954 | 0,10 | 1,68 |
| *Levofloxacin* | 156 | 0,00 | 35,26 | 166 | 0,00 | 52,41 | 203 | 0,00 | 46,31 | 192 | 0,00 | 38,02 | 190 | 0,00 | 29,47 | 907 | 0,00 | 40,24 |
| *Linezolid ** | 152 | 0,00 | 0,00 | 165 | 0,00 | 0,61 | 217 | 0,00 | 1,38 | 202 | 0,00 | 1,98 | 214 | 0,00 | 1,40 | 950 | 0,00 | 1,16 |
| *Streptomicyn* | 153 | 0,00 | 40,52 | 166 | 0,00 | 46,39 | 202 | 0,00 | 40,59 | 190 | 0,00 | 35,26 | 188 | 0,00 | 25,53 | 899 | 0,00 | 37,37 |
| *Teicoplanin ** | 156 | 0,00 | 1,28 | 169 | 0,00 | 1,78 | 216 | 0,00 | 1,39 | 203 | 0,00 | 1,48 | 211 | 0,00 | 3,32 | 955 | 0,00 | 1,88 |
| *Tigecycline ** | 155 | 0,00 | 0,00 | 166 | 0,00 | 0,00 | 214 | 0,00 | 0,47 | 203 | 0,00 | 1,48 | 211 | 0,00 | 3,32 | 949 | 0,00 | 1,16 |
| *Vancomycin ** | 155 | 0,00 | 3,87 | 169 | 0,00 | 2,96 | 216 | 0,00 | 1,85 | 201 | 0,00 | 1,49 | 211 | 0,00 | 3,79 | 952 | 0,00 | 2,73 |

| ***Klebsiella* *pneumoniae*** | **2015** | | | **2016** | | | **2017** | | | **2018** | | | **2019** | | | **2015-2019** | | |
| --- | --- | --- | --- | --- | --- | --- | --- | --- | --- | --- | --- | --- | --- | --- | --- | --- | --- | --- |
| **ANTIBIOTIC** | **Assays n.** | **I%** | **R%** | **Assays n.** | **I%** | **R%** | **Assays n.** | **I%** | **R%** | **Assays n.** | **I%** | **R%** | **Assays n.** | **I%** | **R%** | **Assays n.** | **I%** | **R%** |
| *Amikacin ** | 177 | 2,82 | 13,56 | 209 | 0,96 | 12,92 | 207 | 7,25 | 10,14 | 309 | 6,80 | 3,24 | 296 | 1,01 | 14,19 | 1198 | 3,84 | 10,35 |
| *Amoxicillin/*  *clavulanic acid* | 178 | 0,00 | 46,07 | 239 | 0,00 | 51,88 | 257 | 0,00 | 52,53 | 367 | 0,00 | 66,76 | 346 | 0,00 | 35,26 | 1387 | 0,00 | 51,05 |
| *Ampicillin* | 132 | 0,00 | 100,00 | 192 | 0,00 | 100,00 | 219 | 0,00 | 98,17 | 296 | 0,00 | 100,00 | 150 | 0,00 | 96,00 | 989 | 0,00 | 98,99 |
| *Cefepime* | 178 | 6,74 | 33,15 | 239 | 12,13 | 39,75 | 308 | 9,74 | 30,19 | 363 | 29,48 | 32,23 | 371 | 5,39 | 46,36 | 1459 | 13,57 | 36,74 |
| *Cefotaxime* | 178 | 0,56 | 45,51 | 239 | 0,42 | 54,81 | 262 | 0,76 | 50,38 | 367 | 0,54 | 62,40 | 394 | 1,02 | 51,78 | 1440 | 0,69 | 53,96 |
| *Ceftazidime* | 178 | 4,49 | 35,96 | 239 | 2,93 | 49,79 | 257 | 2,72 | 48,64 | 366 | 3,01 | 59,29 | 398 | 3,52 | 47,99 | 1438 | 3,27 | 49,79 |
| *Ciprofloxacin* | 178 | 5,06 | 41,01 | 239 | 5,44 | 46,44 | 257 | 2,72 | 51,36 | 367 | 1,36 | 62,94 | 398 | 2,26 | 54,02 | 1439 | 2,99 | 52,95 |
| *Ertapenem* | 162 | 0,00 | 21,60 | 239 | 0,00 | 28,45 | 256 | 0,00 | 29,30 | 366 | 1,64 | 19,95 | 397 | 1,51 | 25,94 | 1420 | 0,85 | 24,93 |
| *Fosfomicyn c/G6P* | 178 | 0,00 | 24,72 | 239 | 0,00 | 27,20 | 256 | 0,00 | 31,25 | 366 | 0,00 | 29,23 | 395 | 0,00 | 35,70 | 1434 | 0,00 | 30,47 |
| *Gentamicin* | 178 | 12,92 | 24,72 | 239 | 12,13 | 30,96 | 257 | 8,17 | 32,68 | 367 | 2,18 | 46,05 | 398 | 1,26 | 32,66 | 1439 | 5,98 | 34,82 |
| *Imipenem ** | 177 | 10,73 | 17,51 | 228 | 10,53 | 17,11 | 257 | 4,67 | 14,01 | 362 | 7,46 | 11,33 | 373 | 4,02 | 18,23 | 1397 | 6,94 | 15,39 |
| *Meropenem ** | 178 | 1,69 | 25,84 | 239 | 2,51 | 24,69 | 256 | 2,34 | 24,61 | 366 | 1,91 | 15,85 | 396 | 3,03 | 20,45 | 1435 | 2,37 | 21,39 |
| *Norfloxacin* | 132 | 0,00 | 49,24 | 171 | 0,58 | 53,80 | 126 | 0,79 | 44,44 | 230 | 0,43 | 62,17 | 34 | 0,00 | 61,76 | 693 | 0,43 | 54,40 |
| *Piperacillin/tazobactam* | 177 | 7,34 | 44,07 | 239 | 9,62 | 47,70 | 255 | 11,76 | 39,22 | 367 | 26,70 | 35,42 | 396 | 10,86 | 39,90 | 1434 | 14,44 | 40,45 |
| *Trimet/sulfam* | 178 | 0,00 | 39,89 | 239 | 0,00 | 50,63 | 256 | 0,39 | 51,95 | 367 | 1,09 | 65,12 | 398 | 0,00 | 41,96 | 1438 | 0,35 | 50,83 |

| ***Escherichia coli*** | **2015** | | | **2016** | | | **2017** | | | **2018** | | | **2019** | | | **2015-2019** | | |
| --- | --- | --- | --- | --- | --- | --- | --- | --- | --- | --- | --- | --- | --- | --- | --- | --- | --- | --- |
| **ANTIBIOTIC** | **Assays n.** | **I%** | **R%** | **Assays n.** | **I%** | **R%** | **Assays n.** | **I%** | **R%** | **Assays n.** | **I%** | **R%** | **Assays n.** | **I%** | **R%** | **Assays n.** | **I%** | **R%** |
| *Amikacin ** | 597 | 6,53 | 0,34 | 618 | 4,85 | 0,32 | 646 | 19,35 | 0,46 | 710 | 6,20 | 0,14 | 795 | 2,39 | 0,50 | 3366 | 7,64 | 0,36 |
| *Amoxicillin/*  *clavulanic acid* | 690 | 0,00 | 31,59 | 918 | 0,00 | 38,89 | 929 | 0,00 | 44,56 | 1053 | 0,00 | 43,21 | 1277 | 0,00 | 28,27 | 4867 | 0,00 | 37,09 |
| *Ampicillin* | 633 | 0,00 | 67,93 | 811 | 0,00 | 67,57 | 904 | 0,00 | 71,46 | 950 | 0,00 | 71,26 | 621 | 0,00 | 64,90 | 3919 | 0,00 | 69,00 |
| *Cefepime* | 689 | 8,85 | 18,72 | 919 | 10,45 | 15,78 | 929 | 10,33 | 18,95 | 1043 | 9,49 | 19,37 | 1218 | 6,90 | 22,41 | 4798 | 9,09 | 19,28 |
| *Cefotaxime* | 690 | 0,43 | 30,87 | 919 | 0,44 | 25,57 | 930 | 0,75 | 29,46 | 1053 | 1,04 | 28,96 | 1246 | 0,80 | 30,50 | 4838 | 0,72 | 29,08 |
| *Ceftazidime* | 690 | 7,25 | 22,03 | 919 | 6,53 | 18,06 | 930 | 7,20 | 20,65 | 1054 | 7,69 | 20,11 | 1260 | 5,79 | 23,57 | 4853 | 6,82 | 21,00 |
| *Ciprofloxacin* | 690 | 0,43 | 49,57 | 919 | 0,44 | 42,87 | 930 | 1,51 | 45,27 | 1054 | 2,18 | 46,39 | 1258 | 5,88 | 47,30 | 4851 | 2,43 | 46,20 |
| *Ertapenem ** | 689 | 0,15 | 0,87 | 918 | 0,87 | 5,23 | 929 | 1,08 | 5,38 | 1050 | 0,38 | 6,00 | 1258 | 0,32 | 4,77 | 4844 | 0,56 | 4,69 |
| *Fosfomycin c/G6P ** | 690 | 0,00 | 4,64 | 918 | 0,00 | 6,10 | 930 | 0,00 | 4,62 | 1052 | 0,00 | 6,65 | 1251 | 0,00 | 5,68 | 4841 | 0,00 | 5,62 |
| *Gentamicin* | 690 | 0,43 | 18,41 | 919 | 0,98 | 18,39 | 930 | 1,29 | 22,04 | 1054 | 1,61 | 20,30 | 1260 | 1,43 | 17,70 | 4853 | 1,22 | 19,33 |
| *Imipenem ** | 597 | 0,00 | 0,17 | 831 | 2,53 | 0,72 | 929 | 1,72 | 0,86 | 1044 | 3,07 | 1,05 | 1222 | 2,37 | 1,06 | 4623 | 2,12 | 0,84 |
| *Meropenem ** | 689 | 0,29 | 0,73 | 918 | 1,74 | 1,85 | 926 | 2,59 | 1,19 | 1051 | 1,62 | 1,43 | 1258 | 1,51 | 1,43 | 4842 | 1,61 | 1,36 |
| *Nitrofurantoin ** | 688 | 0,00 | 3,05 | 911 | 0,00 | 3,40 | 913 | 0,00 | 2,85 | 1043 | 0,00 | 4,12 | 1195 | 0,00 | 4,44 | 4750 | 0,00 | 3,66 |
| *Norfloxacin* | 548 | 0,73 | 53,10 | 583 | 0,34 | 47,86 | 549 | 0,55 | 53,37 | 588 | 1,02 | 48,47 | 93 | 1,08 | 53,76 | 2361 | 0,68 | 50,74 |
| *Piperacillin/tazobactam ** | 687 | 0,58 | 12,52 | 915 | 1,53 | 9,95 | 927 | 2,16 | 10,36 | 1046 | 3,15 | 7,84 | 1250 | 1,92 | 11,04 | 4825 | 1,97 | 10,22 |
| *Trimet/sulfam* | 690 | 0,00 | 39,28 | 918 | 0,11 | 39,65 | 930 | 0,22 | 36,45 | 1053 | 0,19 | 38,56 | 1260 | 0,32 | 36,11 | 4851 | 0,19 | 37,83 |

| ***Proteus mirabilis*** | **2015** | | | **2016** | | | **2017** | | | **2018** | | | **2019** | | | **2015-2019** | | |
| --- | --- | --- | --- | --- | --- | --- | --- | --- | --- | --- | --- | --- | --- | --- | --- | --- | --- | --- |
| **ANTIBIOTIC** | **Assays n.** | **I%** | **R%** | **Assays n.** | **I%** | **R%** | **Assays n.** | **I%** | **R%** | **Assays n.** | **I%** | **R%** | **Assays n.** | **I%** | **R%** | **Assays n.** | **I %** | **R %** |
| *Amikacin ** | 76 | 0,00 | 2,63 | 104 | 1,92 | 0,96 | 97 | 14,43 | 4,12 | 111 | 5,41 | 0,90 | 108 | 4,63 | 2,78 | 496 | 5,44 | 2,22 |
| *Amoxicillin/*  *clavulanic acid* | 52 | 0,00 | 0,00 | 61 | 0,00 | 0,00 | 58 | 0,00 | 10,34 | 129 | 0,00 | 36,43 | 133 | 0,00 | 21,80 | 433 | 0,00 | 18,94 |
| *Ampicillin* | 58 | 0,00 | 56,90 | 86 | 0,00 | 68,60 | 84 | 0,00 | 59,52 | 100 | 0,00 | 66,00 | 31 | 0,00 | 48,39 | 359 | 0,00 | 62,12 |
| *Cefepime ** | 85 | 42,35 | 3,53 | 115 | 27,83 | 3,48 | 109 | 22,02 | 4,59 | 129 | 17,83 | 6,98 | 110 | 13,64 | 7,27 | 548 | 23,72 | 5,29 |
| *Cefotaxime* | 85 | 1,18 | 47,06 | 115 | 0,87 | 46,09 | 108 | 0,00 | 49,07 | 133 | 0,75 | 30,83 | 125 | 0,00 | 33,60 | 566 | 0,53 | 40,46 |
| *Ceftazidime* | 85 | 2,35 | 44,71 | 115 | 3,48 | 43,48 | 109 | 0,92 | 47,71 | 133 | 2,26 | 29,32 | 126 | 1,59 | 27,78 | 568 | 2,11 | 37,68 |
| *Ciprofloxacin* | 85 | 1,18 | 50,59 | 115 | 4,35 | 57,39 | 109 | 0,92 | 56,88 | 133 | 1,50 | 43,61 | 126 | 0,00 | 48,41 | 568 | 1,58 | 51,06 |
| *Ertapenem ** | 82 | 1,22 | 0,00 | 115 | 0,87 | 6,09 | 109 | 0,00 | 4,59 | 132 | 1,52 | 5,30 | 126 | 0,79 | 7,94 | 564 | 0,89 | 5,14 |
| *Fosfomicyn c/G6P* | 85 | 0,00 | 52,94 | 115 | 0,00 | 49,57 | 109 | 0,00 | 45,87 | 132 | 0,00 | 32,58 | 125 | 0,00 | 33,60 | 566 | 0,00 | 37,18 |
| *Gentamicin* | 85 | 0,00 | 23,53 | 115 | 4,35 | 23,48 | 109 | 2,75 | 28,44 | 132 | 7,58 | 21,21 | 126 | 6,35 | 26,19 | 567 | 4,59 | 24,51 |
| *Meropenem ** | 85 | 0,00 | 1,18 | 115 | 2,61 | 0,87 | 109 | 0,92 | 0,92 | 132 | 1,52 | 0,00 | 125 | 1,60 | 2,40 | 566 | 1,41 | 1,06 |
| *Norfloxacin* | 48 | 0,00 | 47,92 | 78 | 0,00 | 64,10 | 64 | 0,00 | 48,44 | 75 | 1,33 | 42,67 | 9 | 0,00 | 33,33 | 274 | 0,36 | 50,73 |
| *Piperacillin/tazobactam ** | 85 | 10,59 | 9,41 | 112 | 9,82 | 8,93 | 107 | 7,48 | 4,67 | 132 | 10,61 | 1,52 | 125 | 5,60 | 2,40 | 561 | 8,73 | 4,99 |
| *Trimet/sulfam* | 85 | 0,00 | 49,41 | 115 | 0,00 | 57,39 | 109 | 0,00 | 65,14 | 133 | 2,26 | 48,12 | 126 | 0,79 | 53,17 | 568 | 0,70 | 54,58 |

* = low percentage of resistance

| I% = percentage of intermediate |
| --- |
| R% = percentage of resistance  Gentamicin and Streptomycin used for both Gram-positive are used as “High Level Sinergy”. |
| Trimet/sulfam= Trimetoprim/Sulfamethoxazole |
